# Waste Management with the Use of Heuristic Algorithms and Internet of Things Technology

**DOI:** 10.3390/s22228786

**Published:** 2022-11-14

**Authors:** Anna Burduk, Dagmara Łapczyńska, Joanna Kochańska, Kamil Musiał, Dorota Więcek, Ivan Kuric

**Affiliations:** 1 Faculty of Mechanical Engineering, Wroclaw University of Science and Technology, 50-370 Wrocław, Poland; 2 Faculty of Mechanical Engineering and Computer Science, University of Bielsko-Biała, 43-300 Bielsko-Biała, Poland; 3 Faculty of Mechanical Engineering, University of Žilina, 010 26 Žilina, Slovakia

**Keywords:** waste management, logistics management optimization, heuristic methods, IMPACT IoT platform, Tabu search, genetic algorithm

## Abstract

Studies have been performed to improve the process of waste management. They were fulfilled by changing the base of waste logistics management using a combination of intelligent algorithms and the IMPACT IoT platform instead of a human factor. The research was carried out on the example of real data with respect to waste management in a given area. The proposed solution includes a program that simulates the filling of specific waste containers located in various areas. The determined aspects are inconveniences on the routes, affecting the time of moving between the receiving points and the distances between the containers. The variability of the speed and intensity of the containers filling up over time is an additional factor taken into account. The proposed methods yielded the performance of the control of the containers’ filling status in real time, which apparently results in the possibility of a reaction to the current demand just in time. The proposed solution enables the improvement of the waste logistics management process, including avoiding the too-frequent emptying of containers or overfilling them. The combination of the device prototype, the simulation program, and the developed algorithms opens the possibility for further research in the smart city and optimization areas.

## 1. Introduction

Waste management is one of the most important problems in modern civilization [1,2]. The emergence of a huge amount of waste is becoming an increasingly serious problem on a global scale due to dynamic economic and technical developments, resulting in a constantly growing population [3,4,5,6]. The problem is complex and requires a solution based on a comprehensive approach but due to its interdisciplinary nature, there is a need to conduct research in various scientific fields, from chemistry and biology, through to management and IT [7].

Waste management includes the collection, transport, processing, recycling or disposal, and monitoring of waste materials [3]. One of the issues in the area of waste collection is logistics management, including the planning of vehicle routes based on demand at points served in a given area. The decision-making process includes determining the handling time of the reception points, planning routes, and choosing the number and capacity of vehicles. Due to the human factor influence on current decision-making processes, as well as the inability to rely on real-time data, overfilling one container or unnecessarily emptying others occurs very often. This results not only in the esthetic discomfort of residents but, above all, it can cause environmental pollution. The scale of the analyzed problem was the motivation to research the possibilities of supporting decision-making processes in the logistics management of emptying waste containers using modern methods for this purpose [8,9,10].

The aim of this study was to improve the process of waste management by planning routes for garbage trucks based on the real demand of potential stop locations, referring to the vehicle routing problem (VRP). This was fulfilled by changing the base of waste logistics management using a combination of computing tools and intelligent methods instead of the human factor. Data gathering and processing were supported by tools and technology, such as infrared sensors, the intelligent management platform for all connected things (IMPACT platform) and the Internet of Things (IoT) technology [11]. Heuristic methods (Tabu search and genetic algorithm) were used to generate the solutions. In addition, a simulation program written for the needs of the work has been used for test performing and solution verification. The paper includes a literature review (Section 2), the description of heuristic methods used in the improvement process and the proposed solution to the problem (Section 3), as well as all the results (Section 4) and conclusions (Section 5).

## 2. Literature Review

The Internet of Things (IoT) enables the coupling of digital and physical objects using communication technology. It introduces a future vision where computing systems, users, and objects cooperate for convenience and economic benefits [12]. For example, these include low-powered wireless devices, such as radio-frequency identification (RFID) tags [13,14], home automation, industrial automation, medical aids, intelligent energy management, smart grids, and mobile healthcare [15], or new services for citizens, public administration, and companies [16,17]. Because of their centralized architecture, IoT systems are vulnerable to issues, such as the risk of infrastructure paralysis [18], problems with distributing regular software to all devices as a result of an exponential growth of the IoT [19], or a lack of trust due to closed source code [20]. There are a lot of examples of using the intelligent methods in the improvement of the waste management process [21,22]. The heuristic ones are particularly widely used in various improvement problems [7,23,24]. Although they do not guarantee the best solution is found, these types of algorithms are successfully used in transport tasks [25,26] and scheduling problems [27,28]. Heuristic methods are especially supportive in the decision-making process, where the human factor influences the effectiveness of the whole process [29]. There are also numerous examples of heuristic methods’ implementation in waste management tasks [30,31,32,33] and waste-collection vehicles’ routing problems [24]. There are algorithms that are applied most often, such as genetic algorithms [34,35,36] and ant colony algorithms [37,38]. In this paper, the heuristic methods of Tabu search and genetic algorithms were used to solve the problem of routing optimization and proper waste collection. These methods were chosen because they yield analyses of various factors of decision making and find solutions that are near to the optimal one [28] in a relatively short time.

Recent research in the area of waste management process improvement includes:

- programs and models used to determine the best locations of waste containers [6],
- sensor technology where waste information is collected from the smart bin and transmitted to an online platform where citizens can access and check the availability of the compartments scattered around a city [21],
- analysis of the technology used to support the transmission of the filling level of waste containers [22].

The solution proposed in this paper is more complex—it not only uses the sensors and online platform to monitor and report waste level, but it also monitors the intelligent algorithms that calculate the routes and selects vehicles with an appropriate capacity for them, excluding the human factor in the entire process.

## 3. Materials and Methods

### 3.1. Heuristic Methods Used in Waste Management Improvement

The Tabu search algorithm was used because of its easy implementation in transportation tasks, combined with the high quality of generated solutions [39,40,41,42,43,44]. It has a deterministic character, and it belongs to a group of local search algorithms. The idea of Tabu search is based on searching the space created by all possible solutions with a particular sequence of movements. Among them, there are forbidden movements called taboo. The algorithm stores information about proven solutions as a Tabu list (TL), completed by avoiding oscillation around the local optimum.

Genetic algorithms (GAs) are, as opposed to Tabu search, non-deterministic and they belong to a group of stochastic algorithms. This algorithm was chosen because of its specifics—GAs may be successfully implemented in many types of problems, including various kinds of vehicle routing problems (VRP). Furthermore, GAs have a set of modifiable parameters to adjust the algorithm in order to achieve high-quality results in the required time. GAs are a powerful tool in solving complex problems such as waste management. The basic principle of their functioning is based on the imitation of biological processes, namely natural selection and heredity processes. In addition, it is free from the essential constraints imposed by strong assumptions about the search space, i.e., continuity, existence of derivatives and modality of the objective function [45,46,47,48].

### 3.2. Waste Management Process

The data analyzed in the case study were gathered and compiled in cooperation with a company providing waste-collection services in one of the bigger cities in Lesser Poland Voivodeship that specified the following problem. The waste-collection system in the area of over 12.3 km^2^ was analyzed, covering 343 single-family houses. Containers were located for the specific types of household waste at each of the collection points. Residents had a various number of containers for a given type of waste. The situation is presented in Table 1.

If necessary, residents can have more than one container for a given type of waste. Waste exports are based on a weekly schedule that is constant for the area under analysis. However, residents have the option of deciding how often to export individual types of waste based on their estimated needs by choosing from several possible pickup days. The demand for waste disposal of the houses is different, depending on family size, the ages of its members, their lifestyle etc. This also affects the types of waste generated. The demand of each house can also change over time due to going on vacation, having guests, and depending on the frequency in which trash is taken out by a family member. Other important factors are the day of the week and the current time of year (i.e., Christmas, holidays, etc.). There was selected data about the location of all containers, their quantity, type, and their capacity. All the information about the current waste management system (among others, the number and type of vehicles in the considered area, the frequency in which the containers were emptied, and the observations and problems of logistic employees) were also analyzed.

**Figure 1:**
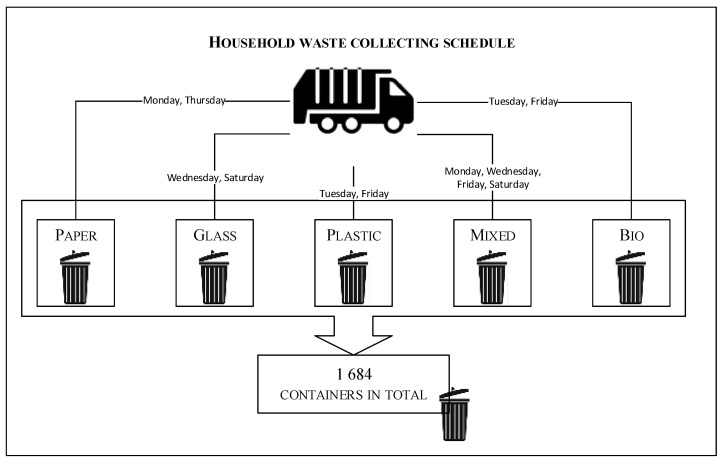
Diagram of household-waste-collection schedule.

The most popular combination was to have one container for each type of waste, including biodegradable garbage. A total of 269 residents decided to work this way, but 54 residents did not have a bio container. Although they should have one, it is assumed that the container for mixed waste is used instead. There were 20 residents who had more containers for mixed or plastic garbage. The company in charge of taking out the trash gathers each type of waste twice a week and the mixed waste is collected four times a week (Figure 1).

The residents purchase a service to collect the garbage according to the schedule above. For example, they can choose to have paper collected each Monday, glass collected every second Saturday, and mixed waste collected twice a week, depending on their plans and needs.

According to the opinions of the residents, 144 were satisfied with such a system and 199 said that something should be changed or optimized. In order to minimize both the logistics costs incurred by the company and the export charges incurred by the residents, while not allowing container reloading, the use technology that enables controlling demand in real time was proposed. The most common problem is inadequate planning in which the company takes out an almost empty or overfilled container. In addition, undesirable situations, such as pouring garbage into a container belonging to another household, were observed. The high prices of the service, combined with cheating and a lack of trust, means that the majority of the owners lock their containers, which need to be opened with a key, the moment they dispose of their garbage, which is what people would like to avoid. The company suffers because of a lack of current information about the fill level of the containers, which makes the company work in a really chaotic way.

### 3.3. The Solution to the Problem

The solution proposed in this paper is a system that charges residents for the volume of their garbage, guaranteeing that containers stay supplied and no longer charging for the number of containers being emptied, as the situation had been up to that moment. The company will be able to collect data about the fill level of containers and adjust its schedule to visit the specific residence on time with the use of sensors installed in the containers. Citizens will not have to choose a plan of garbage collection and will not pay for half empty containers anymore. The expected effect of the solution is a strengthening of company competitiveness by decreasing service-providing costs and giving residents conviction that they are not wasting their money.

The project assumes that all the containers are equipped with sensors reporting the fill level as often as necessary. Based on this information, the company can plan and adjust their collection routes to current needs. Communication can be realized using M2M communication or the Wi-Fi network.

With the use of sensors and the IMPACT platform delivered by Nokia Networks, a test container with a connection system was built. Sample data was gathered and sent using two types of technology:

1. A test M2M SIM card and private LTE network;
2. A Wi-Fi module that the sensor is equipped into and a standard Wi-Fi network.

The proposed methodology included the following steps:

Step 1. Problem formulation—Formulation of the problem and variables, as well as the external conditions and constraints concerning the collection process, and the defining of the objective criterion.

Step 2. Prototyping—Development of a prototype of a waste-tank-emptying system.

Step 3. Heuristic algorithms—Selection and development of algorithms to support the container-emptying system.

Step 4. Results—Conducting tests and analyzing and comparing the obtained results.

Step 5. Algorithms comparison—Conducting additional simulations and comparing the algorithms.

These steps are discussed in detail later in the paper.

Step 1. Problem formulation

A specific container [c] is represented by an integer number defining the type of waste stored in it. Each container also has a defined fulfillment [f] up to 110%, its volume [v] and binary flag [o] reporting if the container is overloaded.

The filling of the container in time takes place according to the specific fulfillment function [fun] with the random element defined according to the company’s advice and observations. Furthermore, the function takes into consideration the fact that during the weekend, social activity and waste production may differ compared with weekdays. The fulfillment function may be the same for various containers. The distance matrix [d] between the containers is determined for route time calculations. The distance between the containers belonging to one household is equal to zero.

During planning, the current statuses of the containers that should be emptied are specified according to their types, the fulfillment level, and their volume. Garbage truck capacity [cap], load time [lt], and the distance matrix are considered in the calculations. To obtain the number of courses [cn] for specific garbage types and truck routes [r], selected containers and distances are taken into consideration. During one trip, only one specific type of garbage can be collected.

There is one optimization constraint (1):

(1) $$\sum_{j=1}^{1684}o_{j}\;\leq \;10,$$

whereas the real-time character of the container fulfillment and the number of acceptable overfilled containers are assumed for 10. The parameters of planning the garbage truck route are shown in Table 2.

The company is equipped with one garbage truck with a fixed capacity of 8 m^3^, and the number of vehicles available is unlimited. According to the assumptions, the total monthly number of routes taken should be minimalized (2).

(2) $$min\sum_{i=1}^{cn}r_{i},$$

In addition, the number of overfilled containers should be minimalized (3).

(3) $$min\sum_{j=1}^{1684}o_{j},$$

The algorithms were tested using a simulation program, and then compared with each other and historical data.

### 3.4. Waste-Container-Emptying System Prototype

Step 2. Prototyping

For the needs of the research, a prototype of a part of the waste-container-emptying system was built using sensors, the IMPACT platform, and IoT technology. A program simulating the filling of specific waste containers located in various parts of the city was written based on company data. The distances between the containers and the inconveniences on the routes affecting the time of moving between the receiving points were determined. The variability of the speed and intensity of the containers filling up over time was also taken into account. By mounting the sensors to the prototype waste containers, it was possible to monitor the level of their filling in real time. The IoT enabled the gathering and exchanging of data from infrared sensors, managed by the IMPACT platform and yielding an adaptation of the emptying process to the real needs of the containers. The producer of the IMPACT IoT platform is Nokia, which enables access to the platform and substantive and technical support in the use of IMPACT on the analyzed problem. The use of the IMPACT platform, in combination with IoT technology, yielded the collection and management of the sensor data flow. The first goal was achieved due to the tools mentioned above, controlling the filling status of the containers in real time, which is the basis for reacting to the current demand.

The question was if whether such a system is conceptually and technically valid, which may be realized in two ways: either with M2M communication, which is more and more widely used in IoT issues; or with the use of a simple Wi-Fi network of the residence. An application simulating the filling of all containers in a decent area was written for the needs of the research. The problem is to manage the car route based on the situation updated with a time. In the next stage, the Tabu search and genetic algorithm were developed in order to select the vehicle routes.

Step 3. Heuristic algorithms

Tabu search in waste management

**Figure 2:**
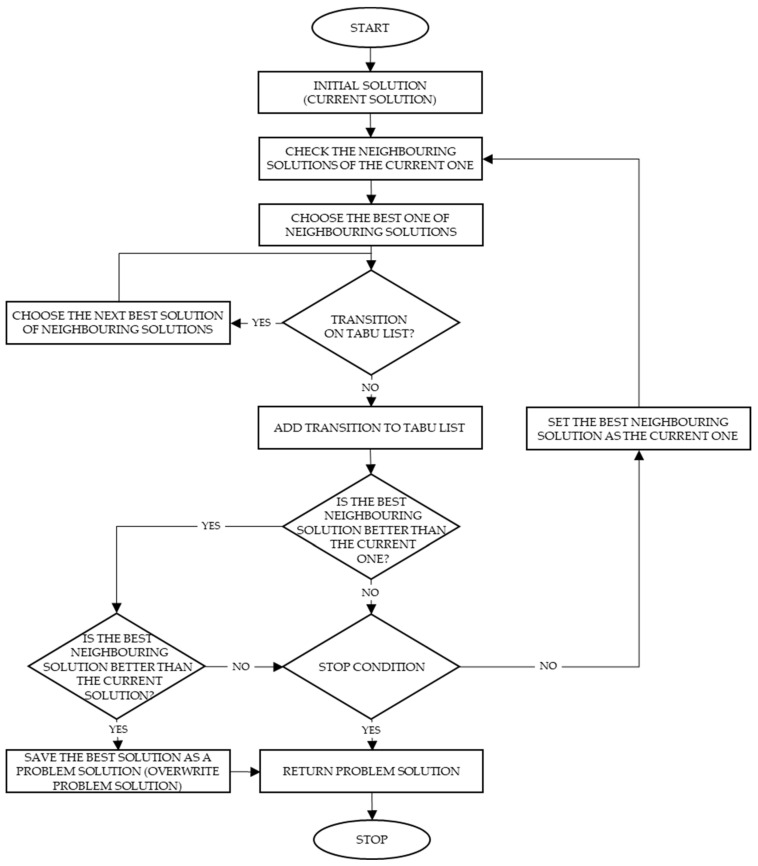
Tabu search diagram.

Optimization problems can be solved using the Tabu search (TS) algorithm, which searches the solution space created by all possible options by means of a specific sequence of movements aimed, at changing the current solution for a better one. The algorithm checks neighboring solutions, taking into account that there are also taboo (forbidden) movements. The algorithm avoids oscillation around the locally optimal solution with the use of information stored in the taboos list (TL). The algorithm does not consider solutions that have been already visited or have violated a rule, thanks to the memory functions. The Tabu search scheme is shown in Figure 2.

The analyzed problem may be presented as a sequence (4):

(4) A B C D E F G…,

in which each letter corresponds to the specific container that has to be reached and the sum of distances between containers should be minimalized.

Checking the neighboring solutions of the current solution

**Figure 3:**
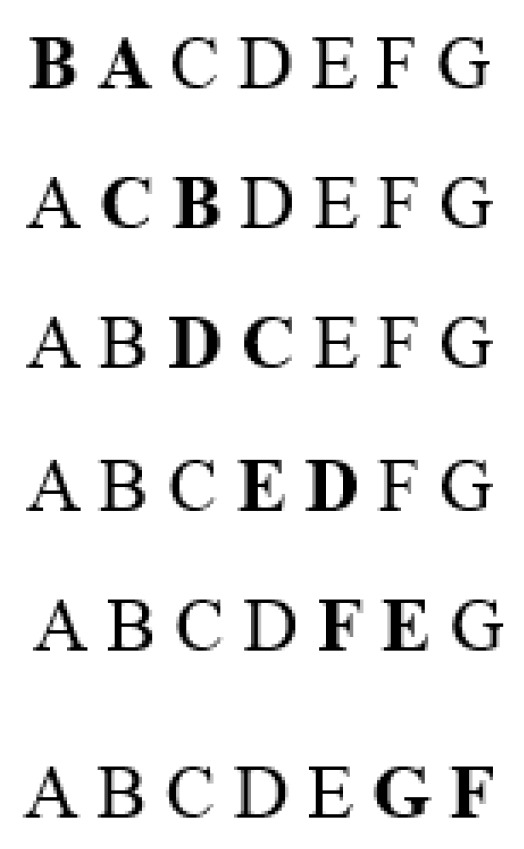
Sequence of the nearest elements in checking the neighboring solutions step.

The algorithm checks all of the neighboring solutions (with one change of the nearest elements in the sequence, Figure 3):

Choosing the best one of the neighboring solutions: the best solution is chosen according to the objective function (minimum sum of distances between containers).

Is the transition on the Tabu list?: In this step, the algorithm checks whether the transition between the current solution and the best solutions has been already proceeded. If so, the best solution is omitted and the next best solution is chosen.

Adding the transition to Tabu List: The algorithm adds the transition to the Tabu list, i.e., the list of prohibited movements (TL). It does not allow the algorithm to be stuck in the locally optimal solution. 

Setting the best neighboring solutions as the current one: The algorithm updates the current solution to start from it in the next iteration.

Saving the best solution as a problem solution/overwriting the problem solution: The Tabu search algorithm allows a transition to be made to the worse solution. The final solution does not have to be the one chosen in the last iteration; therefore, the algorithm updates the problem solution within every iteration.

Genetic algorithm

**Figure 4:**
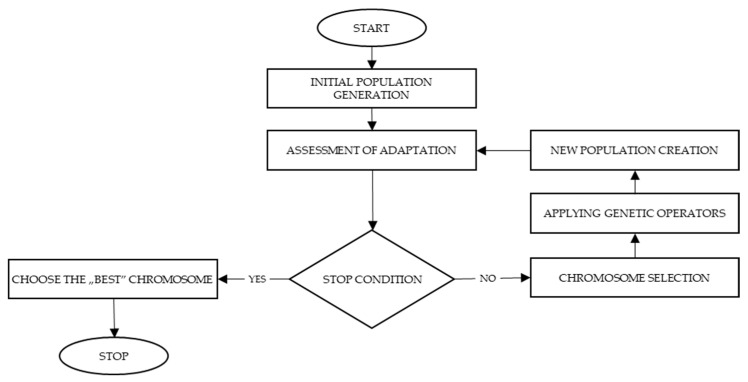
A scheme of genetic algorithm.

The genetic algorithm (GA), which belongs to the class of evolution algorithms, is an example of an algorithm analyzing many samples in each step, in contrast to the Tabu search based on a simple sample analysis. A genetic algorithm diagram is presented in Figure 4.

The genetic algorithm consists of several main operations. The first, which is called initialization, generates a set of initial solutions (an initial population). In the next step, the individuals generated before (initial solutions) are evaluated and selected to designate solutions for further steps. The selected individuals can be reproduced or moved with no changes to the next generation. The reproduction process consists of 2 main activities: crossover (mixing previously determined solutions) and mutation (adding some random changes). In the following case, a time period of 4 weeks (6 working days with 8 h working time and 1 free day) was assumed.

## 4. Results and Discussion

Step 4. Results

The problem taken into consideration is based on a standard travelling salesman problem (TSP), an optimization problem that consists of finding the minimum Hamilton cycle in a full-weighted graph. In practice, it means finding the shortest way that a car should take to reach all mandatory points. Because each route includes collecting only one type of garbage, and containers belonging to a specific house are in the same place (the distance between them is equal to 0), the maximum size of the problem is equal to 343 collecting points, which yields an approximate 2.97 · 10^719^ possible routes. The average route includes 70–100 containers.

A simulation was performed in order to investigate this issue. A private LTE network was configured and sample data were sent from the sensor to the IMPACT platform through the network using LTE and Wi-Fi technology, in cooperation with the telecommunication company concerned. The technological requirements were met. Furthermore, a simulator of container fulfilment was written based on fulfilment functions. Therefore, a trash car receives an update once an hour, and the route can be recalculated.

According to fulfilment functions and company information, three variants were taken into consideration:

- Variant 1—safe, where each container is 80% filled (and the mixed container is 70% filled) and has to be emptied with the nearest route for a specific garbage type and not later than within 2 days.
- Variant 2—average, where each container is 85% filled (and the mixed container is 75% filled) and has to be emptied with the nearest route for a specific garbage type and not later than within 2 days.
- Variant 3—risky, where each container is 90% filled (and the mixed container is 80% filled) and must be emptied with the nearest route for a specific garbage type and not later than within 3 days.

The problem was resolved using both the Tabu search and genetic algorithm for a 4-week period. Table 3 shows that the schedule being followed by the company may be improved.

Unfortunately, because of the high variability in container fulfillment, the adjustment of the optimal number of route trips caused an increase in the number of overfilled containers, which can be noticed in the case of Variant 3. Both the genetic algorithm and the Tabu search provided an acceptable solution. Furthermore, it is impossible to claim clearly which of them is more efficient—it is case related. According to the assumptions, the genetic algorithm provided a better solution and spent one course less than the Tabu search. If it was allowed to overfill up to 45 containers, the Tabu search would have been better. A single calculation takes up to 5 min using a medium-class computer, which is acceptable according to the route update each hour.

In order to enable the company to manage the routes flexibly, while ensuring a reduction in unnecessary export fees, payment for the number of full containers was proposed. The simulations of the process of filling all analyzed containers and emptying them in accordance with the orders of residents was carried out for its validation.

**Figure 5:**
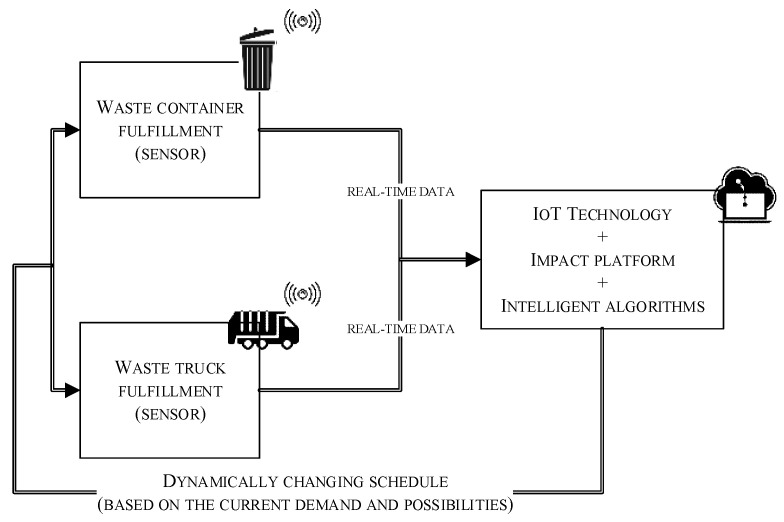
New emptying system diagram.

In the improved system, the company collects data on the current demand at potential pickup points every morning and, using algorithms, calculates preliminary routes for a given day, selecting vehicles with the appropriate capacity for them. The data is downloaded in real time, while the calculations are repeated every hour, taking into account the level of garbage collection at that moment. As a result, the routes can be modified according to the current demand (by changing their fragments and adding or omitting any of the pickup points) and to the vehicle-loading capabilities (Figure 5).

**Figure 6:**
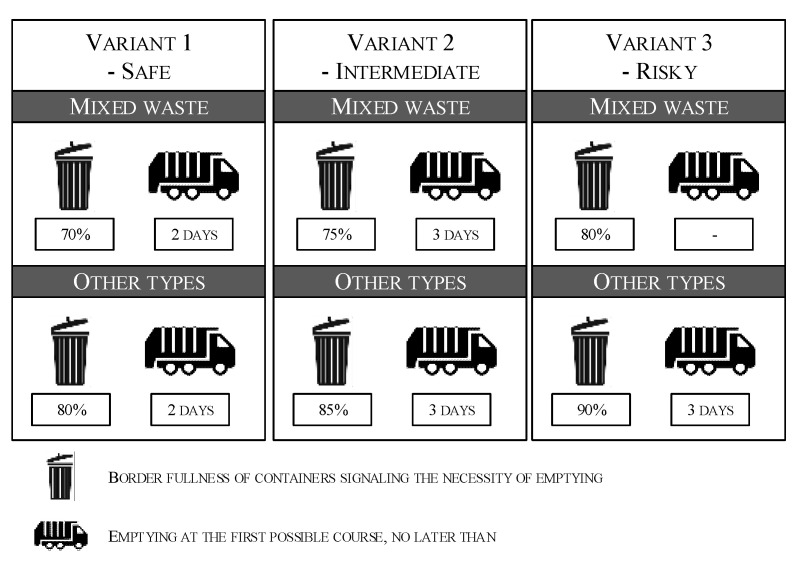
New emptying system—load levels.

Three variants, safe, average, and risky (Figure 6), of the system operation were tested, differing in the level of border fullness of the containers and the maximum time of their emptying.

**Figure 7:**
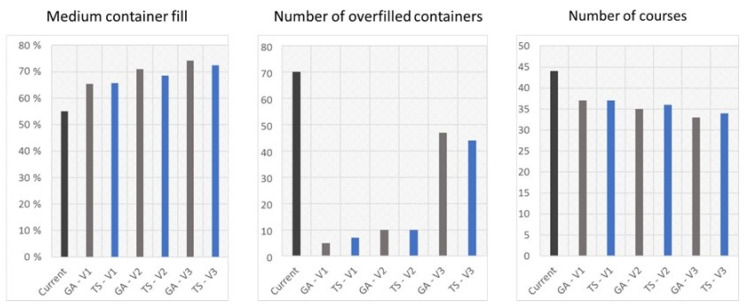
Simulation results for each variant using both algorithms.

The results of the simulations using both algorithms (GA: genetic algorithm; TS: Tabu search) for each variant (VARIANT 1: V1; VARIANT 2: V2; VARIANT 3: V3), compared with the current results, are presented in Figure 7.

The simulations using the presented technology and algorithms have shown that it is possible to reduce the level of container overflow, while reducing the trips performed, thus reducing costs. The results obtained using both algorithms have been compared, and the genetic algorithm was slightly better. The best solution was to use the genetic algorithm in Variant 2.

Step 5. Algorithms comparison

The fulfillment function is presented in Step 4. The results have been prepared with the specific level of difficulty needed to reproduce the real situation presented in the article. For example, the fulfilment function allowed (with certain probability) the whole container to be filled in 2 days, but it did not allow the whole container to be filled in 1 day. In the following steps, additional simulations with various difficulty levels (with 1 being the highest and 8 being the lowest) were performed and Tabu search and genetic algorithms were compared. In these simulations, a 1-week period was taken into consideration, which helped to make single calculations of the algorithms visible. Because of this one-week period, the number of courses was not taken into account.

**Figure 8:**
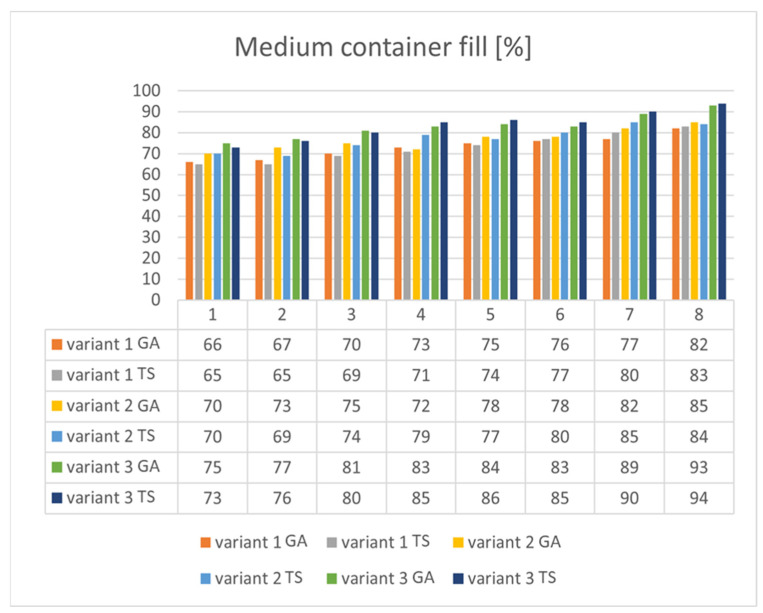
Simulation results of filling a medium container for various difficulty levels (each variant using both algorithms).

**Figure 9:**
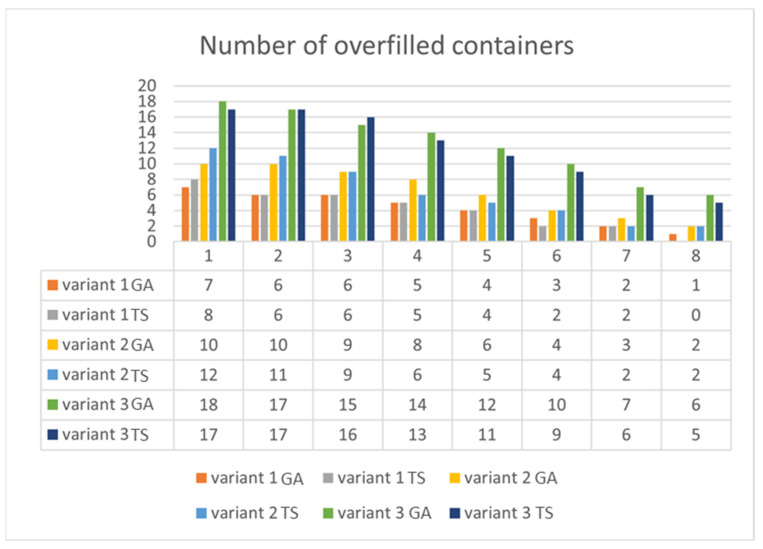
Simulation results of number of overfilled containers for various difficulty levels (each variant using both algorithms).

As seen in Figure 8, it is impossible to arbitrarily say that any algorithm is better. In the cases of Variant 1 and Variant 3, the GA provided a slightly better solution for a higher difficulty of fulfillment function (simulations 1 and 2) and vice versa: Tabu search won for a lower difficulty of fulfillment function (simulations 6–8). In Variant 2, there were significant differences between the algorithms, but it was impossible to notice an unambiguous pattern. In simulation 4, there was a big difference between the algorithms: TS; 79% vs. GA; 72%, which shows a greater stability of solutions for Tabu search. Figure 9 presents various simulations for the number of overfilled containers. It is impossible to notice one general pattern, but for a higher difficulty of fulfillment function (simulations 4–8), it is clearly seen that TS provides a better solution for all three variants.

## 5. Conclusions

**Figure 10:**
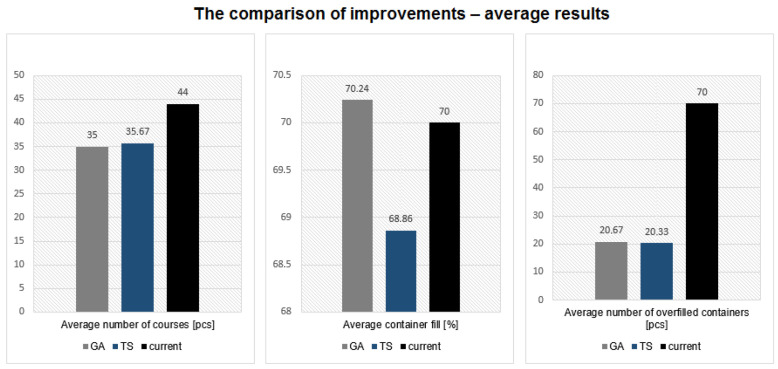
Improvement comparison—average results.

To analyze the results of the improvement, the averages of the data was calculated and compared to each other. The comparisons are shown in Figure 10.

Almost all of the results confirm that the use of the Tabu search and genetic algorithms can be used in the improvement of the waste management area. It is especially visible in the case of the number of overfilled containers—both of the methods decreased this amount for about 50 containers, which is about 43% of the total overfilled containers. This results not only in an increase in the esthetical aspect of the city, but also in the safety, which depends on the amount of waste in the street. The average decrease in the fill level of the containers was not so high. In the case of using the GA, it was a low increase; however, the average number of trips taken was lower than in the current situation at about five trips. This aspect is especially important while looking at waste management as the whole system of transport that need to be conducted almost every day. Additionally, the comparison of TS and GAs for various fulfillment functions shows that Tabu search can provide a better solution in the case of an easy fulfillment function and Variant 3—a high container fulfillment was accepted. In current simulations, it has been noticed that the TS algorithm has greater stability of solutions.

In summary, the aim has been achieved. The proposed methods enabled the performance of controlling the containers’ filling status in real time, which resulted in a reaction to the current demand just in time. The proposed solution yields an improvement in the waste logistics management process, including avoiding the too-frequent emptying of containers or overfilling them. The combination of the prototype of the device, the simulation program, and the developed algorithms opens the possibility for further research in the smart city and optimization areas. While working on this article, the next potential improvements were noticed:

1. The optical sensor placed in the container can be susceptible to pollution. In a further investigation, a weight for each container can be taken into consideration.
2. The company can propose that their customers adjust the container system: to use a different number of containers or different sizes of containers to reduce the number of overfilled ones.
3. The company is going to replace two of their vehicles with newer models and gather new customers because new houses and flats are under construction. Vehicle capacity, adjusted to company needs, together with new route schedules, can bring additional savings.

## Figures and Tables

**Table 1:** Types of waste in containers.

House Type	Houses No.	Container No.						
		1	2	3	4	5	6	7
House type 1	54 pcs	mixed	plastic	paper	glass	x	x	x
House type 2	269 pcs	mixed	plastic	paper	glass	bio	x	x
House type 3	17 pcs	mixed	plastic	plastic	paper	glass	bio	x
House type 4	3 pcs	mixed	mixed	mixed	plastic	paper	glass	bio
Total	1684 containers							

**Table 2:** The parameters of planning the garbage truck route.

Parameter Type	Parameter Mark	Parameter Value
Container type	[c]	[c_1_, c_2_, …, c_1684_]
Fulfillment	[f]	[f_1_, f_2_, …, f_1684_]
Volume	[v]	[v_1_, v_2_, …, v_1684_]
Overfilling	[o]	[o_1_, o_2_, …, o_1684_]
Filling function	[fun]	[fun_1_, fun_2_, …, fun_1684_]
Distance matrix	[d]	[d_1,1_, d_1,2_, …, d_1684,1684_]
Truck capacity	[cap]	8000 litters
Load time	[lt]	120 s
Route number	[cn]	integer positive value
Route	[r]	[r_1_, r_2_, …, r_cn_]

**Table 3:** Variant 1–3 comparison.

No of Trips	Waste Type					Average Container Fill [%]	Overfilled Containers	Sum No. of Courses
	Mixed	Paper	Plastic	Glass	Bio			
company	12	8	8	8	8	50–60	60–80	44
variant 1—GA	10	7	7	6	7	65.42	5	37
variant 1—TS	10	7	7	6	7	65.68	7	37
variant 2—GA	9	7	7	5	7	71.03	10	35
variant 2—TS	9	7	7	6	7	68.43	10	36
variant 3—GA	9	6	6	5	7	74.27	47	33
variant 3—TS	9	7	6	5	7	72.47	44	34

## Data Availability

The data used in the research is not publicly available.
